# Intact CD100–CD72 Interaction Necessary for TCR-Induced T Cell Proliferation

**DOI:** 10.3389/fimmu.2017.00765

**Published:** 2017-06-30

**Authors:** Xiaojun Jiang, Niklas K. Björkström, Espen Melum

**Affiliations:** ^1^Norwegian PSC Research Center, Department of Transplantation Medicine, Division of Surgery, Inflammatory Diseases and Transplantation, Oslo University Hospital Rikshospitalet, Oslo, Norway; ^2^Faculty of Medicine, Institute of Clinical Medicine, University of Oslo, Oslo, Norway; ^3^Research Institute of Internal Medicine, Division of Cancer Medicine, Surgery and Transplantation, Oslo University Hospital Rikshospitalet, Oslo, Norway; ^4^K.G. Jebsen Inflammation Research Centre, Faculty of Medicine, Institute of Clinical Medicine, University of Oslo, Oslo, Norway; ^5^Center for Infectious Medicine, Department of Medicine Huddinge, Karolinska Institutet, Karolinska University Hospital, Stockholm, Sweden

**Keywords:** semaphorin-4D/CD100, CD72, T cell proliferation, antibody blockade, soluble anti-CD100

## Abstract

Targeting CD100 by antibody blockade is a potential therapeutic strategy for cancers, but the functional effects on T cells following blockade of this immune activating molecule are rarely considered. Indeed, CD100 is highly expressed in T cells and anti-CD100 antibodies play a role during T cell proliferation; however, the outcome varies from different studies and the underlying mechanism is still unclear. To address this, monoclonal antibody clones directed against CD100 were evaluated. In their soluble form, four of these antibodies significantly reduced the expansion of T cells in the presence of bead-bound anti-CD3/CD28, either in total peripheral blood mononuclear cell or purified T cell culture systems. Similar inhibition was seen when blocking CD100–CD72 interaction by soluble anti-CD72 instead of anti-CD100 antibodies. Conversely, restoring the interaction by CD72-Fc eliminated the soluble anti-CD100-induced inhibitory effect. Taken together, these results reveal that T cell proliferation is regulated by CD100 *via* interaction with CD72. They further establish an *in vitro* system to evaluate the inhibitory effect of anti-CD100 antibodies on T cells, to which attention should be paid in clinical trials in order to avoid potential side effects.

## Introduction

CD100, also known as semaphorin-4D (SEMA4D), is a transmembrane lymphocyte semaphorin expressed on immune cells that is also overexpressed on various tumors (1–3). It has been reported that the expression levels of CD100, and one of its receptors, plexin-B1 (PLXNB1), are increased in head and neck, prostate, colon, breast, and lung cancers, and the interaction between them provides oncogenic signaling essential for tumor growth and metastasis (4–6). Based on these findings, CD100 has been considered a novel target for monoclonal antibody (mAb)-based therapy for cancer, an approach that has achieved considerable success in recent years (7). Although no definite indication of inhibited tumor progression has been confirmed in ongoing anti-CD100 clinic trails, patients have been reported to exhibit stable disease, and specific blockade of CD100 is promising based on its antitumor activity in mouse models (8, 9). A proposed mechanism of action for anti-CD100-based tumor rejection is the promotion of immune infiltration into the tumor microenvironment (9). However, in this context, it will be important to know the effects of anti-CD100 blockade on the immune cells, and especially on T cells since these are the immune cells displaying the highest expression of CD100 on their surface (10).

CD100 belongs to the semaphorins, which is a family of proteins that are traditionally associated with neuronal development and guidance (11, 12). CD100 was the first immune semaphorin discovered and is abundantly expressed by most hematopoietic cells (13, 14). It shows higher expression levels on T cells compared to other lymphocytes and the expression is further enhanced after cellular activation (1, 15). Following activation, CD100 can be proteolytically cleaved into a soluble form and then functions as an immune regulator by interacting with its receptors in a similar manner as membrane-bound CD100 (16). Three cellular receptors have been described for CD100, PLXNB1, and Plexin-B2 (PLXNB2) expressed by neural and endothelia cells, and CD72 mainly expressed in the immune system (17–19). CD100 signals are involved in T cell priming, antibody production, and cell-to-cell adhesion (20–22).

Originally, a co-stimulatory role for CD100 was proposed for human T cells as anti-CD100 (clone BD16) increased T cell proliferation under some specific conditions, e.g., CD2-induced proliferation in peripheral blood lymphocytes (PBLs), CD2, or CD3-induced proliferations in purified T cells (1, 15, 23). On the other hand, BD16 also strongly inhibited CD3-induced proliferation in PBL and another anti-CD100 antibody (clone A8/BB18) exerted no effect under these experimental conditions (1, 15, 23). Thus, there is a need to investigate the effect of all different clones of anti-CD100 in an optimal stimulating system to help unveil the function of CD100 on T cells.

In the present study, six commercially available mAb clones against CD100 were tested. Four of them were, in their soluble form, found to inhibit T cell proliferation both in total peripheral blood mononuclear cell (PBMC) and in purified T cell culture systems in the presence of beads coated with anti-CD3/CD28. Soluble anti-CD72 blocking showed a similar inhibitory effect. Furthermore, restoring the CD100–CD72 interaction by introducing a CD72-Fc protein into the system eliminated the inhibitory effect induced by soluble anti-CD100. Thus, an intact CD100–CD72 interaction is essential for T cell proliferation.

## Materials and Methods

### Anti-CD100 mAb Clones

See Table 1 for a detailed description of the anti-CD100 mAb clones used in the study.

**Table 1:** General information about anti-CD100 clones used in this study.

Name	Comp.	Clone	Conc. (μg/ml)	Isotype	Immunogen	Storage buffer
Purified Mouse Anti-CD100	BD	30/CD100	250	Mouse IgG1	aa.721-861	With azide^b^
Anti/human CD100 purified	ebioscience	eBio133-1C6	500	Mouse IgM	Not mentioned^a^	With azide^b^
Monoclonal Anti-semaphorin-4D (SEMA4D) antibody produced in mouse	Sigma	3B4	500	IgG1k	aa.115-224	PBS
Anti-SEMA4D antibody	Abcam	A8/BB18	1,000	IgG1	Human PHA activated lymphocytes	PBS
Human SEMA4D monoclonal antibody (mAb)	R&D	Clone 758734	500	Mouse IgG2b	Met22-Arg734	PBS
Human SEMA4D mAb	R&D	Clone 758726	500	Mouse IgG1	Met22-Arg734	PBS

*^a^Not mentioned by the technical data sheet*.

*^b^Isotypes with azide were used for clone eBio133-1C6 and 30/CD100 as controls to exclude the effect caused by storage buffer*.

### PBMCs Isolation and T Cell Purification

Human PBMCs were prepared from healthy blood donors by Ficoll density gradient centrifugation for immediate use or cryopreservation. T cells were purified by FACS sorting. Fresh or thawed PBMCs were stained with PE-anti-CD45 (HI30, BD Biosciences, Franklin Lakes, NJ, USA), APC-anti-CD19 (HIB19, BD Biosciences), and PE-Cy7-anti-CD3 (UCHT1, eBioscience, San Diego, CA, USA). The CD45^+^CD3^+^CD19^−^ population were defined as T cells. In total, 10 different PBMC preparations were used in our study. The numbers of different donors in each experiment were stated in Figure Legends.

### *In Vitro* Culture and Proliferation Assay

Cells were incubated at 37°C, 5% CO_2_ in RPMI 1640 (Gibco, Thermofisher, Waltham, MA, USA) supplemented with 10% FCS (Gibco) and 1% penicillin/streptomycin (Gibco) in 96-well plates. For the proliferation assay, total PBMCs or purified T cells were pre-stained with Carboxyfluorescein 6 succinimidyl ester (CFSE) prior to the culture. CellTrace™ CFSE Cell Proliferation Kit, for flow cytometry (C34554, Thermofisher) was used for CFSE staining. Five million cells (or sometimes a lower number) were stained in 1 ml CFSE staining buffer (2 μM, FCS-free) for 10 min at 37°C. After washing, 0.2 million of CFSE-labeled PBMCs, or 0.1 million of CFSE-labeled T cells, were seeded into a flat 96-well plate. Dynabeads^®^ Human T-Activator CD3/CD28 (11161D, Thermofisher), soluble anti-CD100 (see above), soluble anti-CD72 (3F3, Biolegend, San Diego, CA, USA), CD72-Fc proteins (Recombinant Human CD72 Fc Chimera Protein, R&D, Minneapolis, MN, USA), or matched isotypes were added as indicated. The dilution of CFSE fluorescence was recorded at day 3 and/or day 5 in the FITC channel on the flow cytometer. The bead-to-cell ratio was 1:2 in the whole PBMC culture or 1:1 in purified T cell culture.

### Flow Cytometry

Fresh or thawed PBMCs were stained for cell surface markers with fluorochrome-conjugated antibodies against the following proteins (dilutions indicated): CD3 1:200 (UCHT1, eBioscience), CD19 1:100 (HIB19, BD Biosciences), CD45 1:100 (HI30, BD Biosciences), CD4 1:100 (RPA-T4, eBioscience), CD8a 1:200 (RPA-T8, eBioscience), CD100 1:50 (A8, Biolegend), CD100 1:50 (unconjugated, 133/1C6, eBioscience), mouse IgM 1:100 (secondary ab for anti-CD100, eB121-15F9, eBioscience), mouse IgG 1:100 (secondary ab for other anti-CD100, Sigma-Aldrich), CD72 1:100 (3F3, Biolegend), and dead cell marker (DCM) 1:200 (Zombie NIR™ Fixable Viability Kit, Biolegend). Data were acquired on a FACSVerse^™^ flow cytometer (BD Biosciences) or on a LSRFortessa™ flow cytometer (BD Biosciences) and analyzed using FlowJo analysis software (Treestar, Ashland, OR, USA).

### RNA Sequencing

RNA samples were prepared using SMARTer Ultra Low RNA Kit for Illumina Sequencing (Clontech). Libraries were clustered using cBot and sequenced on HiSeq2500 (HiSeq Control Software 2.2.58/RTA 1.18.64) with a 2 × 126 setup in Rapid High Output mode. Sequence reads were mapped to reference transcriptome TopHat. Gene level abundance was estimated by HTSeq and batch effects were removed by DESeq.

### Statistical Analyses

All statistical analysis was performed using GraphPad Prism version 6 (GraphPad Software, La Jolla, CA, USA), and *P-*values were calculated by two-tailed unpaired Student’s *t*-test. A *P*-value <0.05 was considered statistical significant.

## Results

### Anti-CD100 Inhibits T Cell Proliferation in a Dose-Dependent Manner

**Figure 1:**
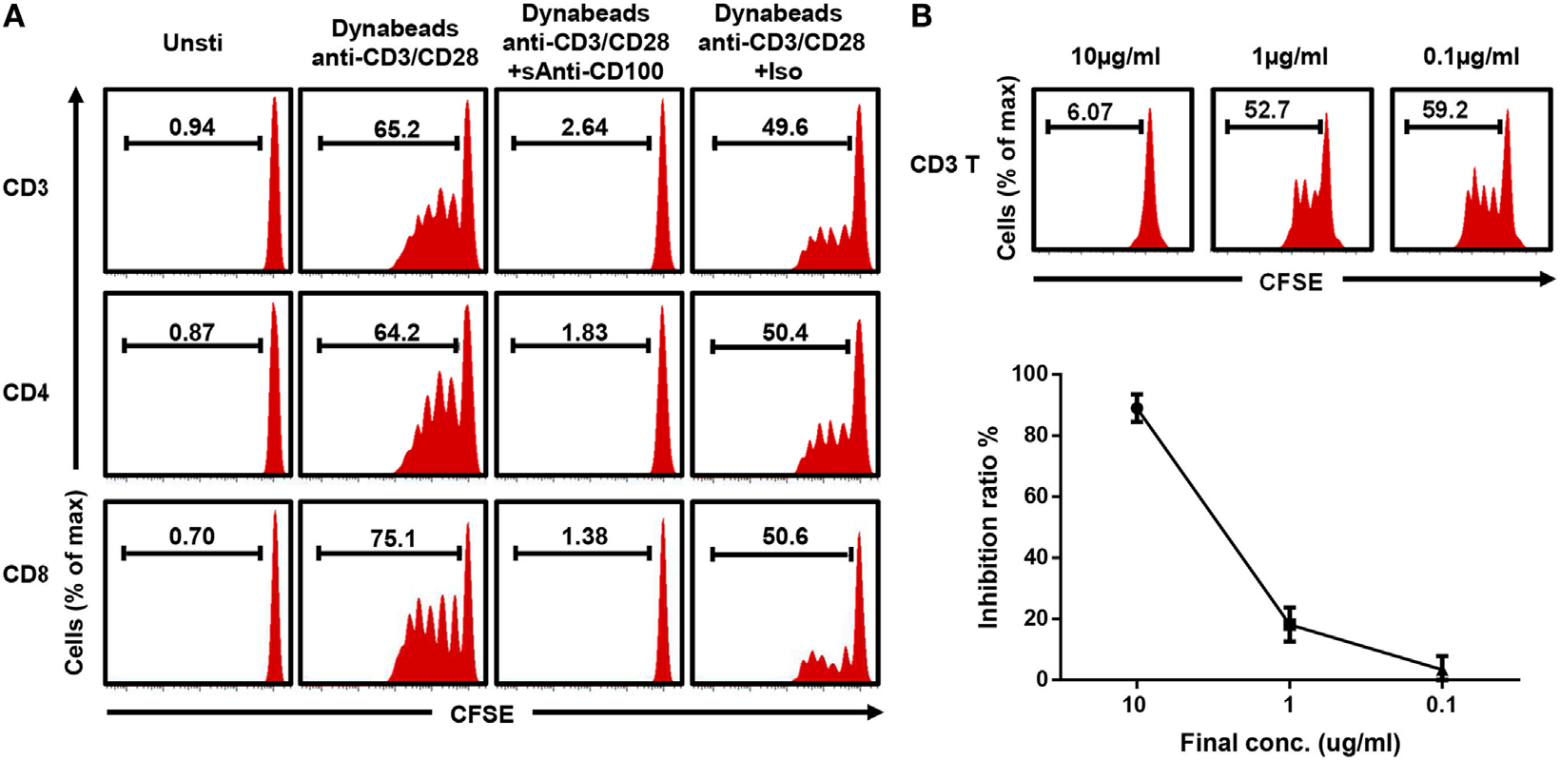
Blocking CD100 inhibits T cell proliferation. **(A)** Representative histograms of total T cells (top), CD4 T cells (middle), and CD8 T cells stained with carboxyfluorescein 6 succinimidyl ester (CFSE) and cultured with Dynabeads anti-CD3/CD28 and soluble anti-CD100 (clone 133-1C6, 10 µg/ml) or isotype control (mouse IgM with azide) after 5 days of culture. The first column shows unstimulated cells. The CFSE^low^ population indicates the proliferating cells. **(B)** Soluble anti-CD100 (clone 133-1C6) at indicated concentrations (10, 1, and 0.1 µg/ml) were added into the culture system (*n* = 4). Inhibition ratio: (proliferated T cells % in isotype control−proliferated T cells % in current group)/proliferated T cells % in isotype control. Titration experiments were repeated twice with peripheral blood mononuclear cells from four different donors.

To establish a stable and repeatable stimulating system to study the role of CD100 in T cell proliferation, we used anti-CD3/CD28-coated beads to stimulate T-cells and first tried to coculture these with soluble anti-CD100 clone 133-1C6. The bead-coated form of this anti-CD100 clone provided the strongest activating signal, measured as interferon gamma secretion, among commercial clones tested in an *in vitro* T cell functional experiment (data not shown). PBMCs were isolated from healthy donors, stained with CFSE, and cultured together with anti-CD3/CD28-coated Dynabeads in the presence of anti-CD100 or isotype control. After 5 days, T cells showed a significant proliferation, which was completely disrupted by adding soluble anti-CD100 clone 133-1C6 at a concentration of 10 µg/ml (Figure 1A). Of note, T cells accounted for over 90% of CFSE^low^ proliferating population (Figure S1A in Supplementary Material), implying that bead-bound anti-CD3/CD28 provides a specific activating signal to T cells even in a mixed PBMC culture. The disrupted proliferation was similar for the CD4 and CD8 subsets of T cells (Figure 1A). As clone 133-1C6 is not optimal for functional assays and azide in the storage buffer can cause an effect in our system (Figure S1B in Supplementary Material), isotype with azide in the same concentration was used to exclude azide-caused effect. The proliferation in the anti-CD100 group was still much lower than that in the matched isotype group (Figure 1A). We reduced the concentration of soluble anti-CD100 and found a clear dose–response relationship (Figure 1B). However, bead-bound anti-CD100 had no clear inhibitory effect on T cell proliferation compared to the soluble form (Figure S2 in Supplementary Material).

### Four Out of Six Antibody Clones Show Similar Inhibition

**Figure 2:**
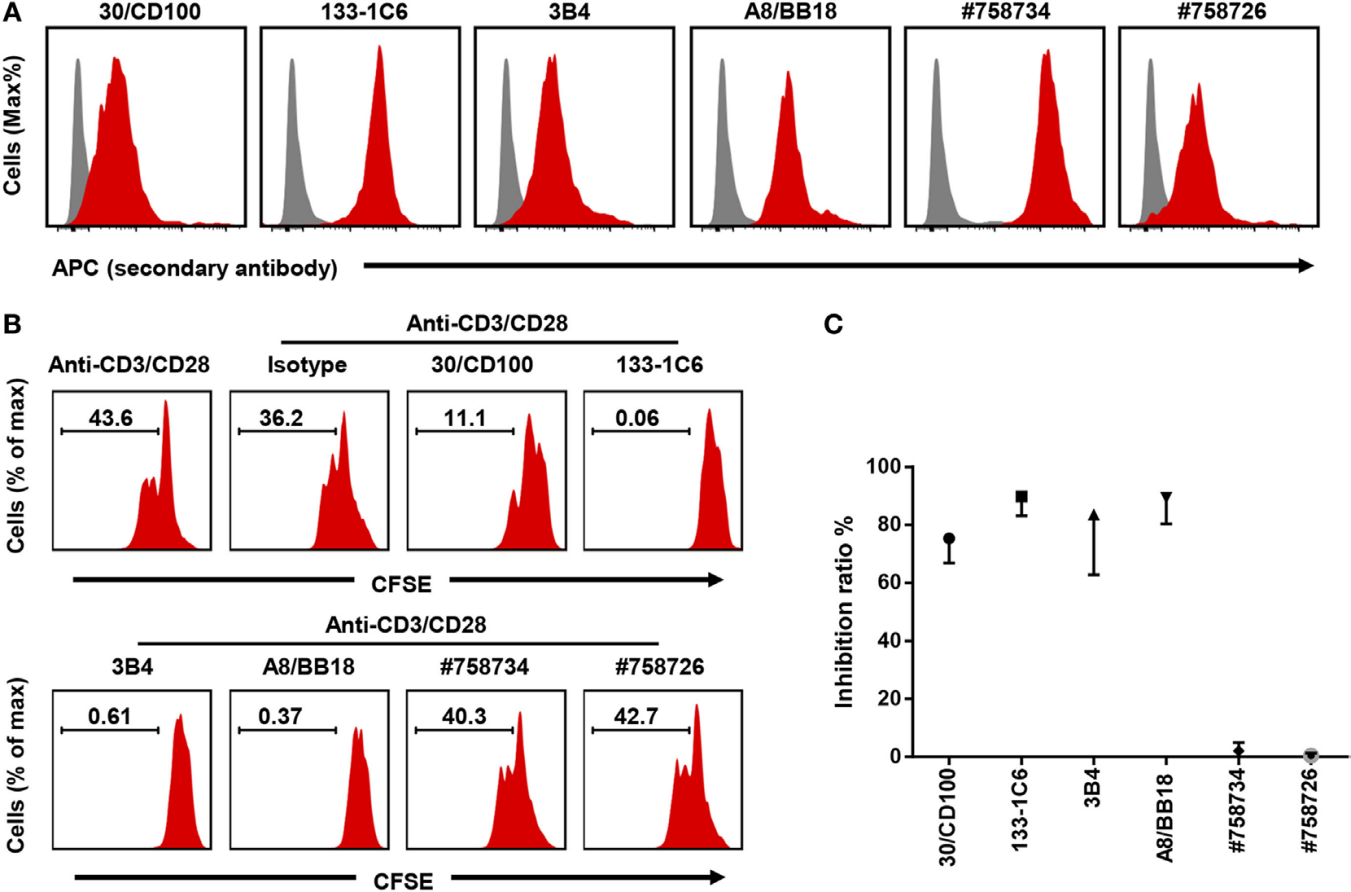
Four out of six anti-CD100 monoclonal antibody clones inhibit T cell proliferation. **(A)** Staining of activated peripheral blood mononuclear cells (PBMCs) with isotype control purified (gray) or different clones of anti-human CD100 purified (red) followed by anti-mouse IgG or IgM APC. PBMCs were stimulated by anti-CD3/CD28. CD3^+^ T cells were used for analysis. Experiments were repeated twice with two different PBMC preparations. **(B)** Total PBMCs pre-stained with Carboxyfluorescein 6 succinimidyl ester (CFSE) and cultured with Dynabeads anti-CD3/CD28 in the presence of indicated soluble anti-CD100 clones, respectively (10 µg/ml). Representative histograms of CFSE fluorescence on T cells after 5 days of culture were shown. Isotype indicates mouse IgM with azide. **(C)** Summary of data showing the inhibition ratio of each clone (*n* = 2–6). Experiments were repeated three times with six PBMC preparations in total.

Having established a robust proliferation assay where CD100 blockade had an inhibitory effect, we next investigated the performance of other commercially available anti-CD100 clones. To this end, five additional anti-CD100 antibody clones were evaluated (30/CD100, 3B4, A8/BB18, #758734, and #758726) (Table 1). First, we checked the binding activity of all the anti-CD100 antibodies to activated T cells using FACS analysis. All these antibodies had good fluorescence signals, suggesting that they all recognize CD100 expressed on cell surface (Figure 2A). Then, their inhibiting effect on T cell proliferation in the total PBMC culture system was analyzed as previously described. Clone 30/CD100, which has been reported as a blocking antibody, showed a weaker inhibition compared to clone 133-1C6 (Figures 2B,C). Clones 3B4 and A8/BB18 inhibited T cell proliferation to a similar but still weaker extent as clone 133-1C6 (Figures 2B,C). Clones #758734 and #758726 did not have any blocking effects. Thus, our data showed an inhibitory effect of four anti-CD100 clones.

### Anti-CD100 Inhibition in a Purified T Cell Culture System

**Figure 3:**
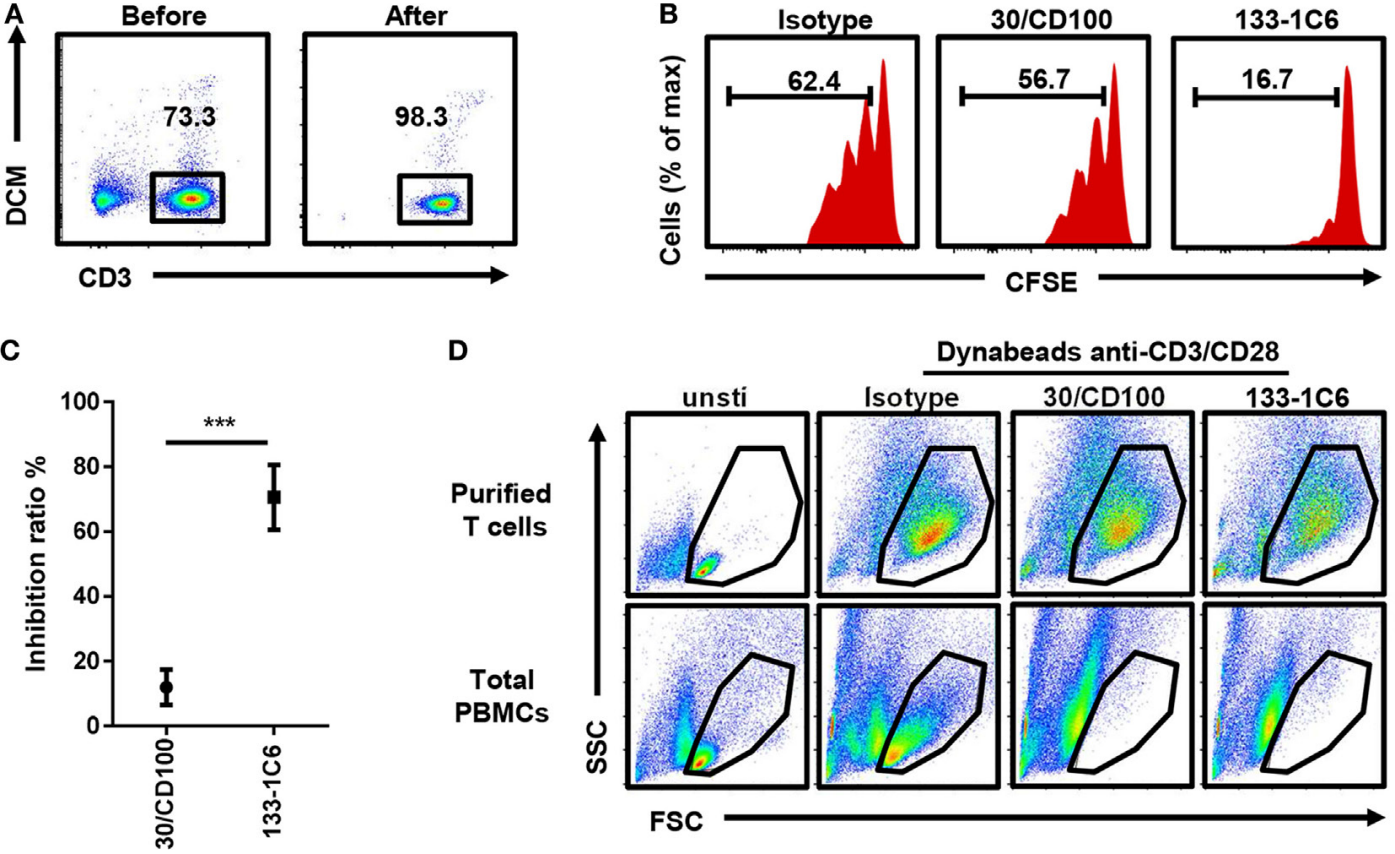
Inhibitory effect of soluble anti-CD100 in purified T cell culture system. **(A)** Peripheral blood mononuclear cells (PBMCs) were stained with anti-CD3 and dead cell marker (DCM). CD3^+^DCM^−^ alive T cells were purified by FACS sorting. **(B)** Purified T cells were stained with Carboxyfluorescein 6 succinimidyl ester (CFSE) and cultured with Dynabeads anti-CD3/CD28 and soluble anti-CD100 clone 30/CD100 or 133-1C6 (10 µg/ml), respectively. CFSE fluorescence on CD3 T cells was analyzed 5 days later. Isotype indicates mouse IgM with azide. **(C)** Inhibition ratio was calculated (*n* = 3, ***P < 0.001). **(D)** FSC/SSC of cultured cells was compared at day 5 in indicated groups. Data were collected from three experiments. Three different individuals were included.

We noticed that almost all proliferated T cells were CD100 positive (Figure S3 in Supplementary Material), raising the possibility that soluble anti-CD100 antibodies inhibit T cell proliferation by a direct effect on T cells or by requiring some helper cells within the PBMCs. To address it, we purified T cells by FACS sorting (Figure 3A). Soluble anti-CD100 (clones 30/CD100 and 133-1C6) still inhibited T cell proliferation in the purified T cell culture system, implying that the inhibiting effect is intrinsic to T cells (Figures 3B,C). Also, in this reductionistic system, clone 133-1C6 showed better inhibitory effect than clone 30/CD100 (Figure 3C). Notably, there was a FSC/SSC population that nearly disappeared in total PBMCs culture system but was retained in the purified T cell culture system (Figure 3D), suggesting that the anti-CD100 effect is more efficient in the PBMC system than in the purified system.

### CD100–CD72 Interaction Is Required for T Cell Proliferation

**Figure 4:**
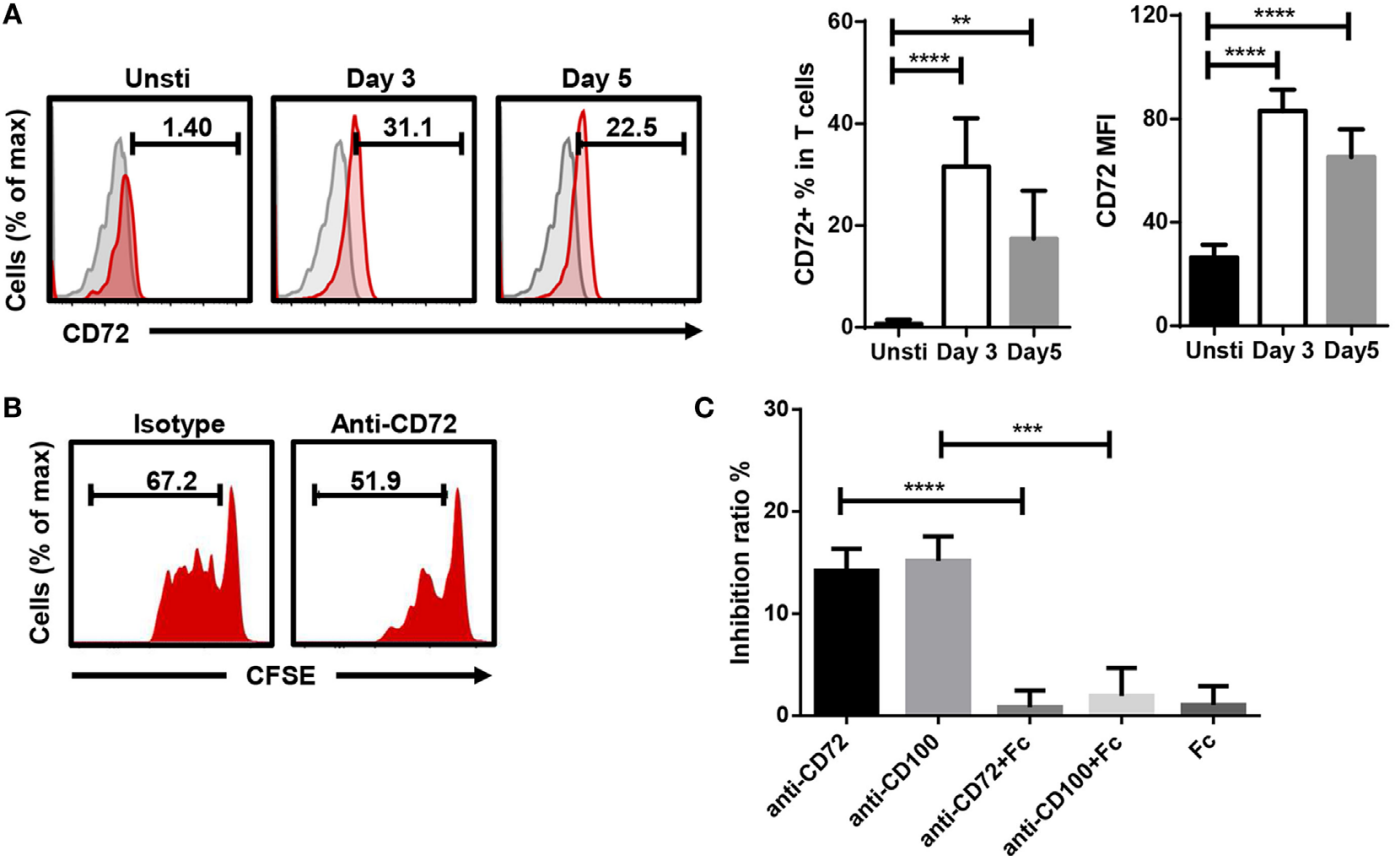
CD100–CD72 interaction is required for T cell proliferation. **(A)** CD72 expression increased on T cells after anti-CD3/CD28 stimulation. Gated on CD3^+^DCM^−^ T cells, gray shadow represents isotype controls. CD72^+^ % in T cells and CD72 MFI (Geom. Mean) of total T cells were subjected to statistical analysis (*n* = 6, **P < 0.01, ****P < 0.0001). **(B)** 5 µg/ml soluble anti-CD72 was added into total peripheral blood mononuclear cell (PBMC) plus Dynabeads anti-CD3/CD28 culture system, Carboxyfluorescein 6 succinimidyl ester (CFSE) fluorescence on CD3 T cells was analyzed 5 days later. **(C)** The inhibiting effect induced by anti-CD100 or anti-CD72 (final conc. 5 µg/ml) was partially eliminated by CD72-Fc (*n* = 4, ***P < 0.001, ****P < 0.0001). The histograms are representative of three independent experiments. PBMCs from six individuals were included in the statistical comparisons.

Soluble anti-CD100 might block the interaction between CD100 and its receptor expressed on T cells. CD100 has three different receptors: PLXNB1, PLXNB2, and CD72. To investigate which receptor was involved, we screened the transcriptional expression of PLXNB1, PLXNB2, and CD72 in CD100^+^-activating T cells by RNA sequencing and noticed the expression level of CD72 was threefold to eightfold higher comparing to that of PLXNB1 and PLXNB2 (Figure S4 in Supplementary Material). Then, we confirmed CD72 protein expression on the surface of T cells by flow-cytometry and found CD72 to be increased after T cell activation (Figure 4A). Soluble anti-CD72 inhibited T cell proliferation in a similar manner as soluble anti-CD100 clone 133-1C6 (Figures 4B,C). Conversely, the inhibiting effect induced by anti-CD100 was eliminated by adding CD72-Fc in the culture system with beads coated with anti-CD3/CD28 and soluble anti-CD100 (Figure 4C). To investigate how the CD100–CD72 interaction was involved in T cell proliferation, we stained for intracellular IL-2 and interferon gamma 24 h after TCR stimulation and did not see any changes when we blocked CD100 (Figure S5A in Supplementary Material). Thus, the inhibited proliferation is probably not due to the changes in TCR secondary events. Also, we found that the inhibitory effect of anti-CD100 was weakened if we used pre-activated PBMCs instead of PBMCs with bead-bound anti-CD3/CD28, implying the effect of CD100–CD72-induced signal to T cell proliferation needs the presence of continuous TCR-signal (Figure S5B in Supplementary Material).

## Discussion

In the present study, we investigated the function of CD100 during T cell proliferation by evaluating a variety of anti-CD100 monoclonal antibodies. Four out of six commercially available anti-CD100 monoclonal antibodies inhibited anti-CD3/CD28-induced T cell proliferation both in total PBMC and in a purified T cell culture system in a CD100–CD72-dependent manner. Only the soluble form of anti-CD100 had the inhibiting effect, while beads coated with anti-CD100 antibodies showed no effect. Compared to previously reported studies, we provided an optimal way to inhibit T cell proliferation by anti-CD100 in our *in vitro* culture systems. In addition, we demonstrate that this inhibiting effect is linked to CD100–CD72 recognition and signaling.

Certain experimental differences exist between the assay established here and previous reports investigating CD100 function. For instance, others have used PBLs in the culture systems (23), whereas we used PBMCs and purified T cells. Actually, the PBLs in other studies were not purified and removed the monocytes, so it should be quite similar to the PBMCs that we used. Previously used activation methods include soluble antibody stimulation. Here, bead-bound anti-CD3/CD28, which provides physiological and highly reproducible activation and expansion of T cells, was used. CD3 and CD28 provide a specific activating signal to T cells, so even in the total PBMC culture system, the vast majority of responding cells were T cells. In addition, we used heat-inactivated serum with a destroyed complement system and mouse anti-human antibodies. Thus, complement-dependent cytotoxicity and antibody-dependent cellular cytotoxicity, two major mechanisms of antibody induced target cell eliminating, are not likely to be involved in our system.

Interestingly, the six anti-CD100 clones investigated gave distinct response patterns. A reason for this might be different binding site of each clone. Anti-CD100 clones BD16 and A8/BB18 were prepared by immunization of BALB/c mice with the human thymic immunizing T cell clone (1, 24), while clones 30/CD100, 133-1C6, 3B4, #758734, and #758726 were obtained by immunization with CD100 peptides for which the binding site is more clear. The binding sites of clones 133-1C6 and A8/BB18 are currently unknown, but it is plausible that these clones bind to the extracellular domain in a manner that interferes with ligand binding due to their blocking effect. Clones #758734 and #758726 bind to extracellular domain of CD100, but they demonstrate no blocking effect. This could be because they either do not block ligand binding or because they actually stimulate CD100.

We found that bead-bound anti-CD100 exhibited no effect on T cell proliferation compared to soluble antibody. Generally, the difference between bead-bound antibodies and soluble antibodies are that the former, if they are activating antibodies, provides stronger signals (25, 26). Since CD72 increased after T cell activation, it is possible that signal induced by CD100–CD72 is at a saturating level in T cells, explaining why we do not observe any additional effect by adding bead-bound anti-CD100. It is also plausible that the bead-coating of anti-CD100 removes the capacity of the antibody to block the interaction between CD100 and CD72. Soluble anti-CD100 blocks CD100–CD72 interaction without providing strong downstream signals, resulting in the inhibition of T cell expansion.

Even though T cells could be divided into CD100^+^ and CD100^−^ populations, we observed that all expanding T cells were CD100^+^, whereas the small CD100^−^ population seemed to lack the capacity to expand. Thus, CD100 is essential for T cell proliferation in our culture system. Interestingly, this crucial role of CD100 is not restricted to T cells. CD100 has also been reported to sustain expansion of B cells as well as myeloid-derived suppressor cells (27, 28). In total PBMCs, there are some other populations expressing receptors of CD100, e.g., Plexin B1 on a subset of DCs, Plexin B2 on monocytes and macrophages, and CD72 on B cells and macrophages (29), which might be associated with the more efficient anti-CD100 inhibitory effect in the total PBMCs system than the purified T cell culture.

Our data suggest that the anti-CD100 inhibits the proliferation of T cells through a blocking effect. It is not likely that anti-CD100 will induce inhibitory signals in T cells as we did not observe any significant inhibition by anti-CD100 coated beads. Also, very little inhibition was seen when adding soluble anti-CD100 to pre-activated PBMCs. We noticed the continuous TCR-stimulating signals were important in our system, but no change was found in TCR-related secondary events, e.g., IL-2 and interferon gamma secretion. Taking into account that CD100 could also affect proliferation in other cell types, CD100 signaling might be directly linked to cell proliferation rather than affecting TCR-induced signaling events, at the same time, the continuous stimulating signal is a prerequisite.

The findings from this study raise concerns regarding the impact anti-CD100 antibodies might have on immune cell function when given to patients in clinical trials. It is hypothesized that antibody neutralization of CD100 inhibits cancer progression by disrupting the CD100–PLXNB1 interaction in tumor tissues and enhancing recruitment of activated monocytes and lymphocytes into the tumor (9, 10, 30, 31). However, if the expansion capacity of the recruited immune cells is inhibited at the same time, the antitumor response will be weakened. Two of the anti-CD100 clones exhibited no inhibition of T cell proliferation, implying that this tentatively negative effect might be avoided by selecting anti-CD100 clones with such features. Thus, it will be important to consider the inhibition effect during clinic trials, to which we provided an optimal system for this to be evaluated in. In addition, CD72-Fc may also protect against this tentative side effect by restoring the CD100–CD72 interaction on T cells, while not disturbing the antitumor effect induced by blocking CD100–PLXNB1 signals.

## Ethics Statement

The study adhered to the guidelines of the Declaration of Helsinki. Written informed consent was obtained from all participants. The ethics applications were approved by the regional Ethics Committee in Norway (2012-286) and the regional Ethical Review Board in Stockholm, Sweden (2013/1449-31/4). There were no immediate negative or positive environmental aspects of the project.

## Author Contributions

XJ conceived and performed experiments, analyzed and interpreted the data, and wrote the manuscript. NB provided human materials, conceived and interpreted experiments, and co-wrote the manuscript. EM designed and interpreted experiments and co-wrote the manuscript.

## Conflict of Interest Statement

The authors declare that the research was conducted in the absence of any commercial or financial relationships that could be construed as a potential conflict of interest.

## Abbreviations

SEMA4D, semaphorin-4d; PLXNB1, plexin-B1; PLXNB2, plexin-B2; mAb, monoclonal antibody; PBLs, peripheral blood lymphocytes; PBMCs, peripheral blood mononuclear cells; CDC, complement-dependent cytotoxicity; ADCC, antibody-dependent cellular cytotoxicity; DCM, dead cell marker.
