# Supplementary material for: Intact CD100–CD72 Interaction Necessary for TCR-Induced T Cell Proliferation
Source: Front Immunol. 2017 Jun 30;8:765. doi: 10.3389/fimmu.2017.00765 (PMC5491939; doi:10.3389/fimmu.2017.00765)
Supplement: Supplementary file 1 [file Presentation_1.PPTX]

## Slide 1
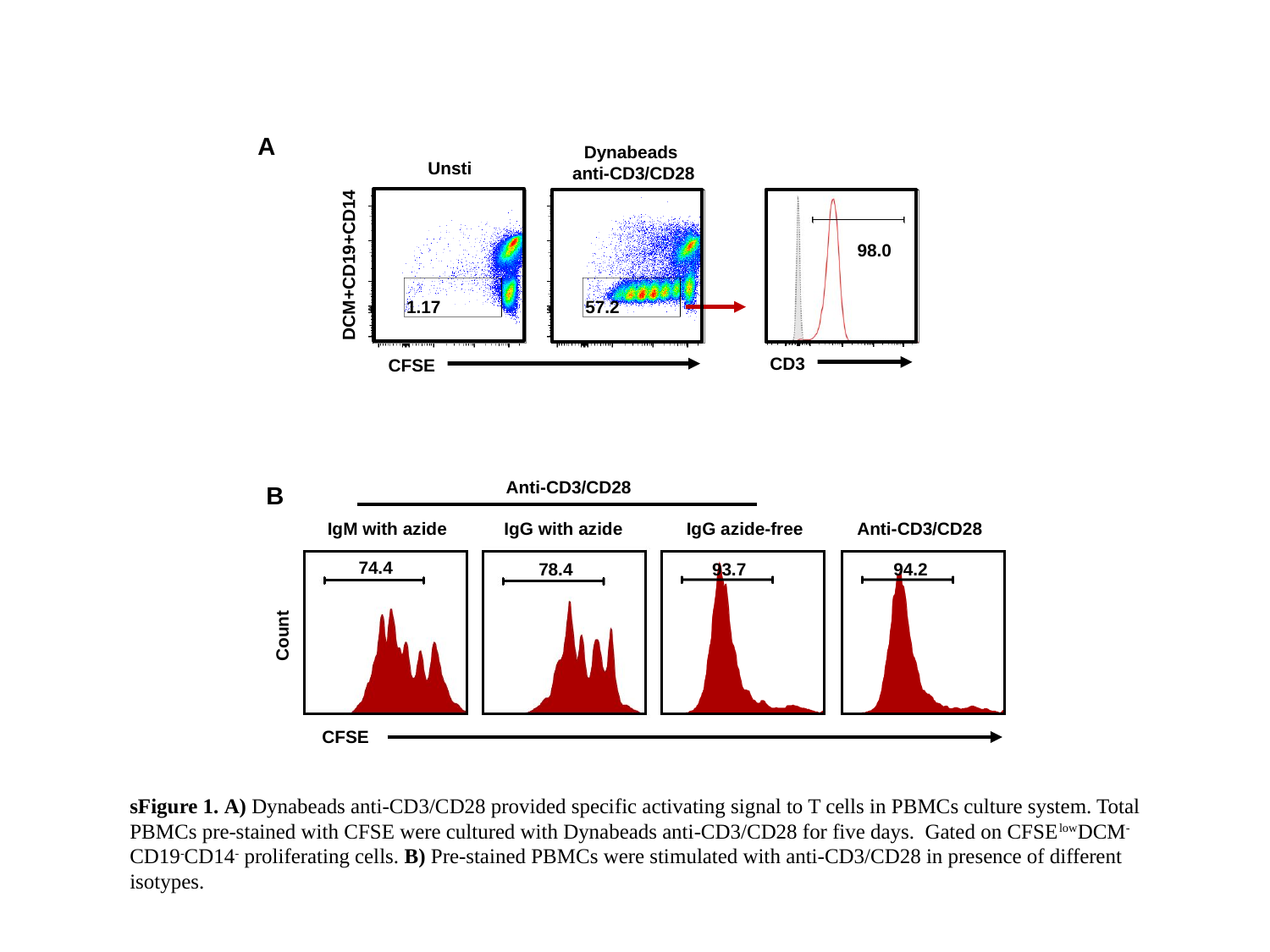

A
Dynabeads
anti-CD3/CD28
Unsti
98.0
DCM+CD19+CD14
1.17
57.2
CD3
CFSE
Anti-CD3/CD28
B
Anti-CD3/CD28
IgM with azide
IgG azide-free
IgG with azide
74.4
78.4
93.7
94.2
Count
CFSE
sFigure 1. A) Dynabeads anti-CD3/CD28 provided specific activating signal to T cells in PBMCs culture system. Total PBMCs pre-stained with CFSE were cultured with Dynabeads anti-CD3/CD28 for five days. Gated on CFSElowDCM-CD19-CD14- proliferating cells. B) Pre-stained PBMCs were stimulated with anti-CD3/CD28 in presence of different isotypes.

## Slide 2
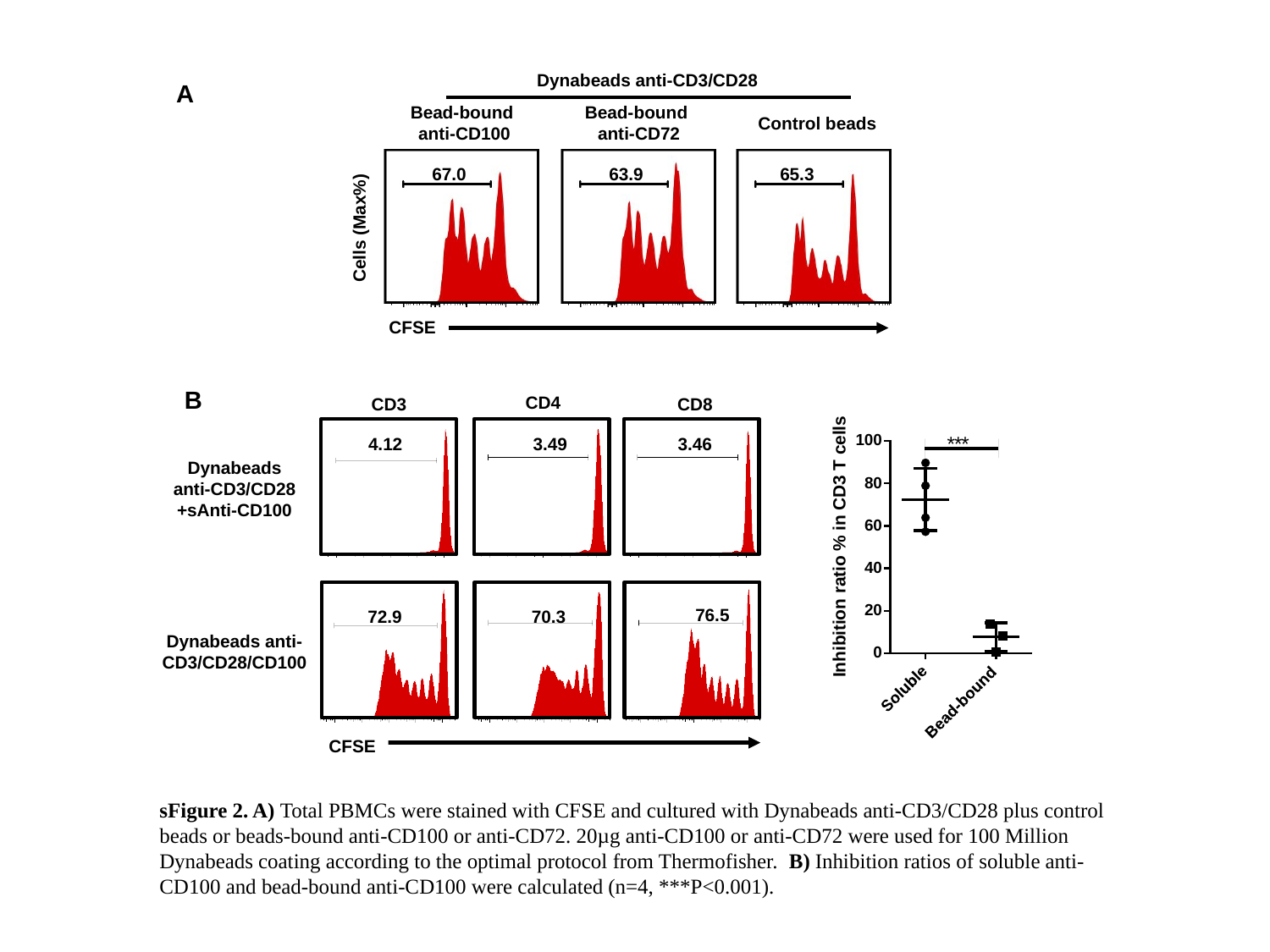

Dynabeads anti-CD3/CD28
A
Bead-bound
anti-CD100
Bead-bound
anti-CD72
Control beads
67.0
63.9
65.3
Cells (Max%)
CFSE
B
CD4
CD8
CD3
4.12
3.49
3.46
Dynabeads
anti-CD3/CD28
+sAnti-CD100
76.5
72.9
70.3
Dynabeads anti-
CD3/CD28/CD100
CFSE
sFigure 2. A) Total PBMCs were stained with CFSE and cultured with Dynabeads anti-CD3/CD28 plus control beads or beads-bound anti-CD100 or anti-CD72. 20µg anti-CD100 or anti-CD72 were used for 100 Million Dynabeads coating according to the optimal protocol from Thermofisher. B) Inhibition ratios of soluble anti-CD100 and bead-bound anti-CD100 were calculated (n=4, ***P<0.001).

## Slide 3
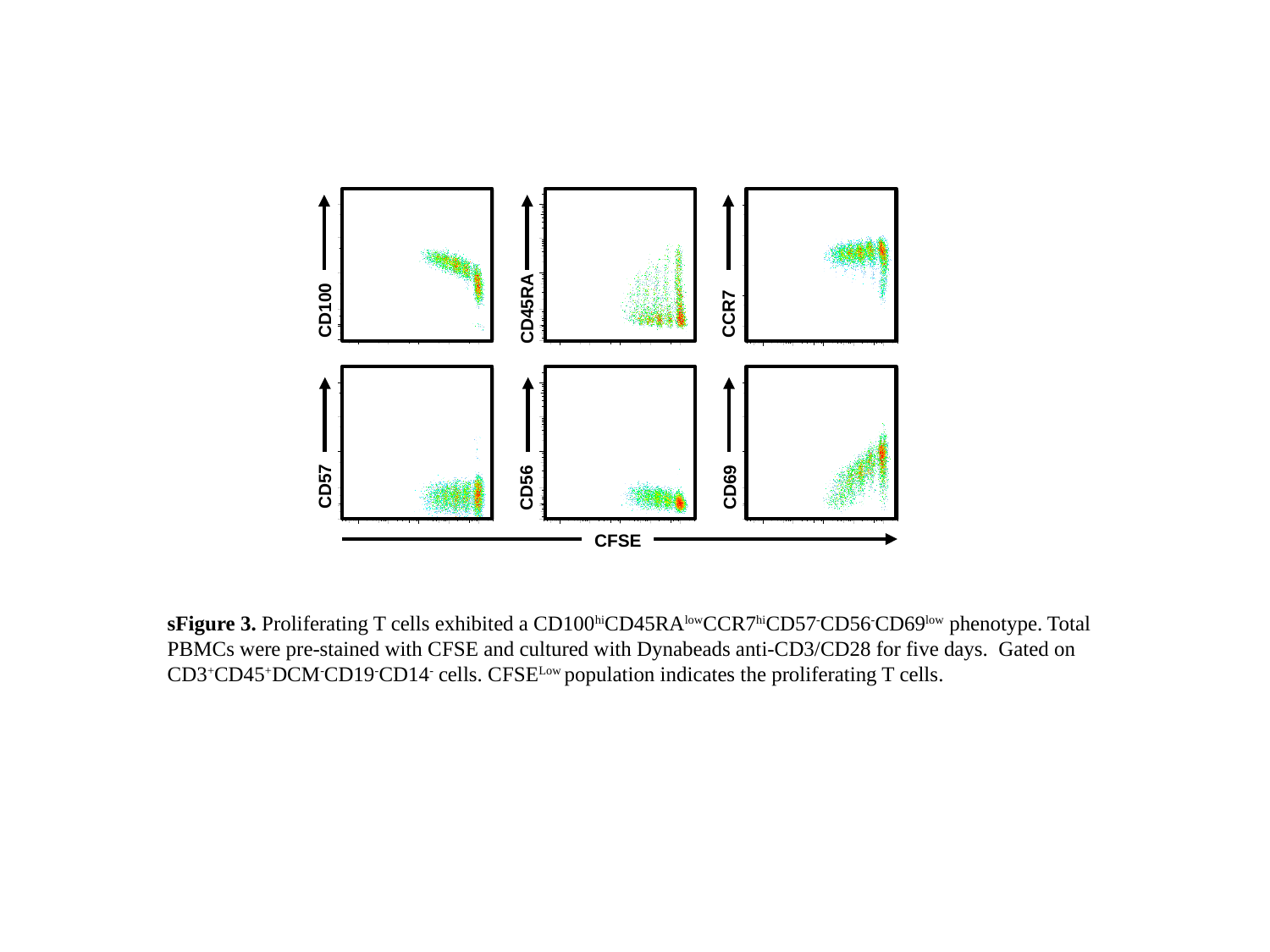

CD45RA
CD100
CCR7
CD57
CD69
CD56
CFSE
sFigure 3. Proliferating T cells exhibited a CD100hiCD45RAlowCCR7hiCD57-CD56-CD69low phenotype. Total PBMCs were pre-stained with CFSE and cultured with Dynabeads anti-CD3/CD28 for five days. Gated on CD3+CD45+DCM-CD19-CD14- cells. CFSELow population indicates the proliferating T cells.

## Slide 4
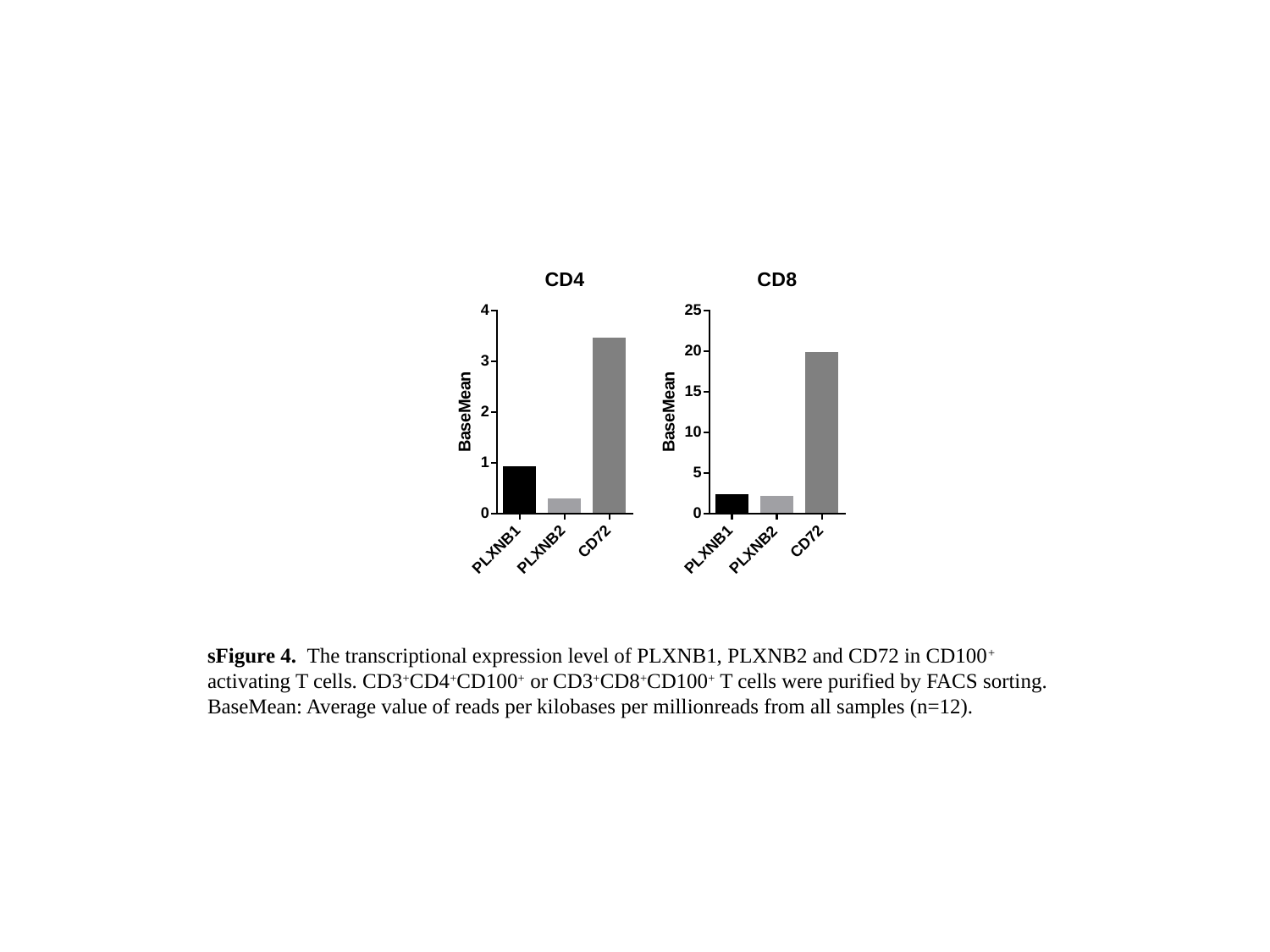

sFigure 4. The transcriptional expression level of PLXNB1, PLXNB2 and CD72 in CD100+ activating T cells. CD3+CD4+CD100+ or CD3+CD8+CD100+ T cells were purified by FACS sorting. BaseMean: Average value of reads per kilobases per millionreads from all samples (n=12).

## Slide 5
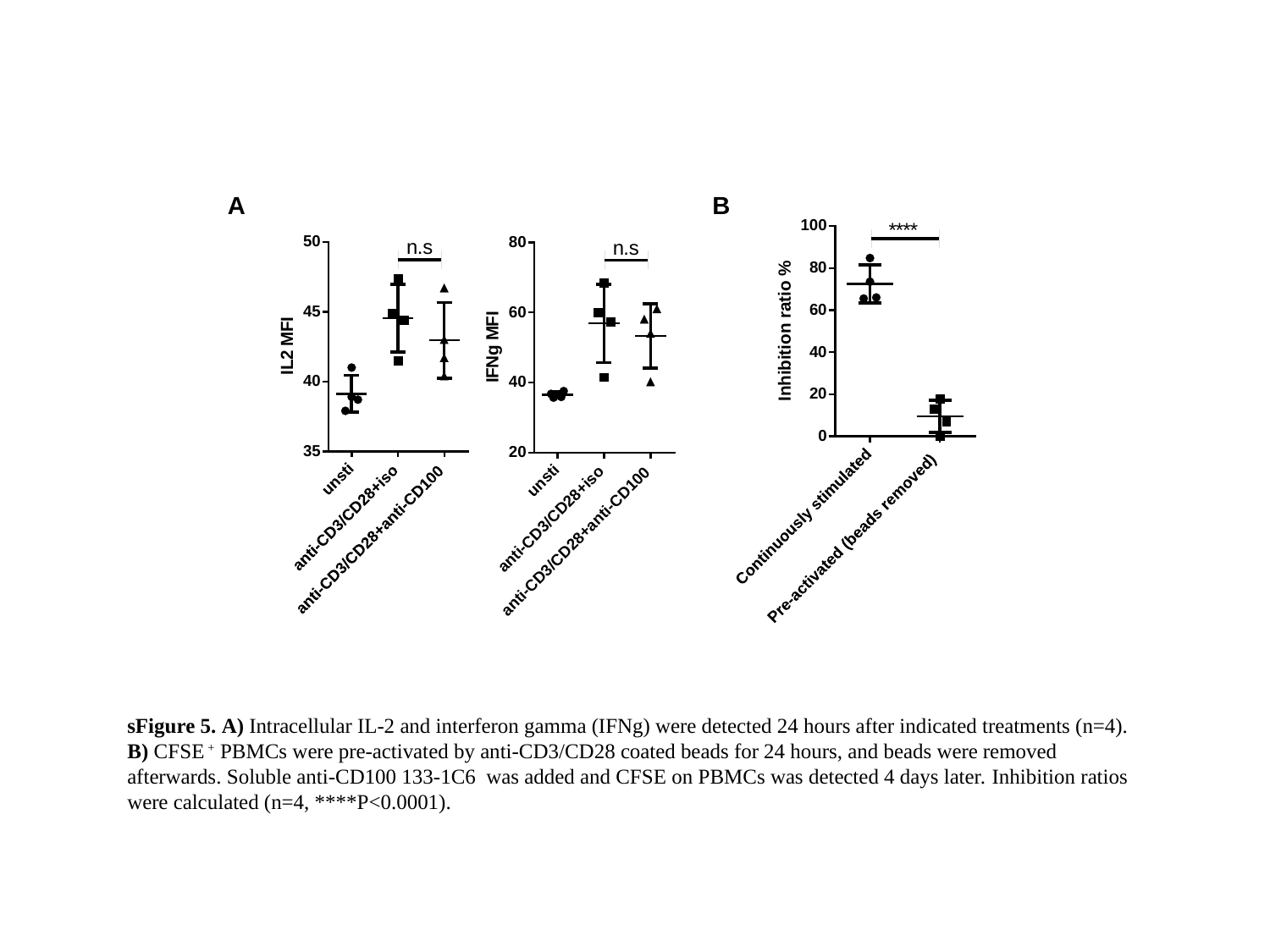

A
B
sFigure 5. A) Intracellular IL-2 and interferon gamma (IFNg) were detected 24 hours after indicated treatments (n=4). B) CFSE + PBMCs were pre-activated by anti-CD3/CD28 coated beads for 24 hours, and beads were removed afterwards. Soluble anti-CD100 133-1C6 was added and CFSE on PBMCs was detected 4 days later. Inhibition ratios were calculated (n=4, ****P<0.0001).
